# An Enhanced SEIR Model for Prediction of COVID-19 with Vaccination Effect

**DOI:** 10.3390/life12050647

**Published:** 2022-04-27

**Authors:** Ramesh Chandra Poonia, Abdul Khader Jilani Saudagar, Abdullah Altameem, Mohammed Alkhathami, Muhammad Badruddin Khan, Mozaherul Hoque Abul Hasanat

**Affiliations:** 1 Department of Computer Science, CHRIST (Deemed to be University), Bangalore 560029, Karnataka, India; rameshcpoonia@gmail.com; 2 Information Systems Department, College of Computer and Information Sciences, Imam Mohammad Ibn Saud Islamic University (IMSIU), Riyadh 11432, Saudi Arabia; altameem@imamu.edu.sa (A.A.); maalkhathami@imamu.edu.sa (M.A.); mbkhan@imamu.edu.sa (M.B.K.); mhhasanat@imamu.edu.sa (M.H.A.H.)

**Keywords:** COVID-19, SEIR model, SEIRV, social distancing, vaccination

## Abstract

Currently, the spread of COVID-19 is running at a constant pace. The current situation is not so alarming, but every pandemic has a history of three waves. Two waves have been seen, and now expecting the third wave. Compartmental models are one of the methods that predict the severity of a pandemic. An enhanced SEIR model is expected to predict the new cases of COVID-19. The proposed model has an additional compartment of vaccination. This proposed model is the SEIRV model that predicts the severity of COVID-19 when the population is vaccinated. The proposed model is simulated with three conditions. The first condition is when social distancing is not incorporated, while the second condition is when social distancing is included. The third one condition is when social distancing is combined when the population is vaccinated. The result shows an epidemic growth rate of about 0.06 per day, and the number of infected people doubles every 10.7 days. Still, with imparting social distancing, the proposed model obtained the value of R_0_ is 1.3. Vaccination of infants and kids will be considered as future work.

## 1. Introduction

Pandemics have plagued humankind for generations. The aftermath of these diseases have a massive impact on the world economies, and the strengths and morals of the heavily impacted nations are compromised. India’s pandemic rate of COVID-19 was so rapid that neither the government nor the people had a chance to respond in a sustainable manner. To combat the impact of the pandemic, the administration must implement timely and soft policies.

It has been almost three years of the world suffering from a global pandemic named COVID-19. COVID-19 is an infectious disease caused by the SARS-CoV-2 virus. This pandemic disease collapsed the world economy and faced a high death rate. As per the report of the World Bank [1], the GDP growth of the year 2020 was −3.405, which was the lowest since the year 1961. According to the World Health Organization [2], 5.31 million people lost their life due to COVID-19, and still new cases are being recorded. COVID-19 is also muting itself with alpha, beta, gamma, and delta versions, which is another alarming situation. Currently the Omicron virus is the latest version of COVID-19 and is rapidly increasing in the world. However, it has still not been verified if Omicron is transmissible or not, or if it is more severe than the Delta mutant. Maintaining social distance and using masks can mitigate the spread of virus. However, vaccination is the only solution that can stop the death rate of COVID-19 pandemic. Vaccination is a biological process that develops acquired immunity to a particular viral disease. The World Health Organization approved 8 vaccines worldwide [3]. These vaccines are shown in Table 1. These vaccines have been approved in various countries and a large number of trials were completed in several of the same countries.

Vaccination drives are being organized in every country. From the total number of the world population, 46.6% are now fully vaccinated. Country-wide vaccination rates are shown below in Table 2 [4].

As shown in Table 2, large number of populations are getting vaccinated rapidly, but still the cases of COVID-19 are increasing. The prime objective of this paper is to predict the COVID-19 cases when the population is fully vaccinated. Basic SIR and SEIR models will be used to predict the COVID-19 cases when the population is fully vaccinated. The subsequent objectives are as follows:

- To enhance SEIR Model with effect different versions of severity.
- To predict the susceptibility, infection and recovered using enhanced model with no social distancing is considered.
- To predict the susceptibility, infection and recovered using enhanced model with social distancing is considered.
- To predict the susceptibility, infection and recovered using enhanced model with social distancing with vaccination is considered.

This paper is organized as such: the introduction, followed by the second section which will cover the background of compartment models, followed by the third section which will cover the related work. Next the fourth section will discuss the proposed model, while the results and discussion are discussed in fifth section, and finally the conclusion and future work is discussed in sixth section.

## 2. Background

The SIR model stands for Susceptible, Infected and Removed [5]. The SIR model is the part of compartment models in epidemiology. This SIR model is used to predict the cases of a pandemic disease, such as dengue, swine flu, plague, and COVID-19, etc. This SIR model spatial-based model works in respective of the total number of population in a particular time stamp. The term “susceptible” means those organisms that can be the host of the infectious disease. The term “infection” means those organisms which are now infected by the disease. The term “removed” means the organism is either recovered from the disease or became deceased from the disease. Mathematical representations of equations are show from Equations (1) to (3):

(1) $$\frac{ds}{dt}=-\frac{\beta IS}{N}\;\;$$

(2) $$\frac{dI}{dt}=\frac{\beta IS}{N}-\gamma I\;$$

(3) $$\frac{dR}{dt}=\gamma I\;\;\;$$

where:

- *N* = total number of population of a geographical location, (*S* + *I* + *R* = *N*)
- *β* is the average number of contacts per person per time
- *γ* is the transition rate assumed to be proportional to the number of infectious individuals

However, this model has some assumptions, such as every individual coming from the population has an equal probability to contract the disease, the total number of death other than from that disease, and new births ignored in this model. The removed persons cannot be re-infected.

The next enhanced model is the SEIR model [6]. Individuals who have been infected but have not yet become contagious have a large latency period. Susceptible-Exposed-Infectious-Removed (SEIR) models are useful in forecasting the trends of occurrence of a disease over a course of time. Thus, it may prove important for epidemiologists to model disease outbreaks. Moreover, it helps us to visualize how a disease will evolve in a population. Further, it categorizes a populace into four categories, namely: Susceptible (*S*), Exposed (*E*), Infectious (*I*), and Removed (*R* or *D*), based on the degree of infection and their potential to transmit the infection. People who are not infected yet but are at a high risk of getting infected are named as susceptible. People who have been infected but are responsible for spreading the infection are said to be exposed. People who are potent disease transmitters are named as infectious (*I*). People who have recovered from illness or are dead, are classified as removed (*R* or *D*). These are neither susceptible, nor do they have the potential to spread the disease. The SEIR model uses the coefficients viz. β to represent the rate of disease exposure, for showing the rate of infection, *γ* for rate of recovery, μ to denote the rate of death, and ε  to represent exposed rate. These individuals are known as exposed in the SEIR model. The mathematical representation of SEIR model is shown from Equations (4) and (5):

(4) $$\frac{dE}{dt}=\frac{\beta IS}{N}-\varepsilon E\;$$

(5) $$\frac{dI}{dt}=\varepsilon E-\gamma I\;\;\;$$

Different versions of SIR model are reviewed. One of the simplest forms of the compartment model is the SIS model, where the individuals cannot develop the hard immunity and individuals are getting infected multiple times. The common flu is the best example of the SIS model. Another significant model is maternal susceptible infectious recovered (MSIR). Other enhanced models are shown below in Table 3.

## 3. Related Work

The latest enhanced SEIR model is the SEIRV model, in which the vaccination compartment is incorporated. Related work of the SEIRV model is shown in this section.

Rabih Ghostine et al. [17] proposed an enhanced SEIQRDV model, which stood for susceptible, exposed, infected, quarantine, recovered, death, and vaccinated. This paper aimed to study the impact of the vaccination rate on COVID-19 spread. The dataset was taken from Saudi Center for Diseases Prevention and Control. The dataset contained different attributes such as deaths, active cases, and recovered cases on a daily record basis. Model parameters are updated through Joint-knKF method. The forecasting has been completed from 1 July to 17 December 2021. The performance of the proposed model is found satisfactory if the RAME value obtained is less than 5%. However, slight changes are observed in RMAE value in the month of June, with a value of 12%, but for the remainder of the month the RMAE value obtained was less than 13%.

Time dependent SEVIS model was proposed by Li et al. [18]; the simulation of the proposed model was completed on the USA dataset starting from 17 March 2020. The results replicated that the number of the infected and recovered individuals would keep increasing at a high rate in the short future. The proposed model predicted that if the average vaccination rate was 1% per day and the likelihood of obtaining immunity after recovery was 50%, the pandemic would be finished by autumn 2021.

The SEIR model was implemented through the patient’s age and the vaccination by Huaixing Li and Jiaoyan [19] Wang. The basic R_0_ is produced. According to their findings, the pace at which vulnerable persons A are recruited significantly impacted the spread of infectious illnesses. As a result, even with vaccines, measures such as travel restrictions and public gathering bans should be implemented for an extended period to maintain the low recruitment rate of vulnerable persons A. This research might aid in the prediction and eradication of infectious illnesses.

Wang et al. [20] presented the age-structured and vaccination-based SEIR model. They assessed the impact of various age-specific vaccination distributions on controlling the COVID-19 outbreak. The vaccination rates V were set at 0.05 percent, 0.1 percent, and 0.15 percent for each instance. They demonstrated that population age structures and social interaction patterns had a major impact on the effectiveness of age-specific immunization programs. In the situation of limited vaccination availability, distinct age-specific priority criteria for the general population must be considered the successfully manage the COVID-19 pandemic. Furthermore, individual countries must design distinct immunization programs based on their population’s age structure and social interaction patterns.

A new SEIRV model was implemented in Sri Lanka by Rajapaksha [21], in which they used a compartmental model to forecast changes in epidemiological indicators. We simulated various vaccination tactics using a dynamic Susceptible-Exposed-Infected-Recovered-Vaccinated (SEIRV) model under a variety of epidemic scenarios. To reduce the sick population as quickly as feasible, at least 45 percent vaccination coverage was essential. The model’s R_0_ was variable and uncontrolled in theory. Parameters such as vaccine effectiveness and vaccination rate may be tweaked alternatively.

**Figure 1:**
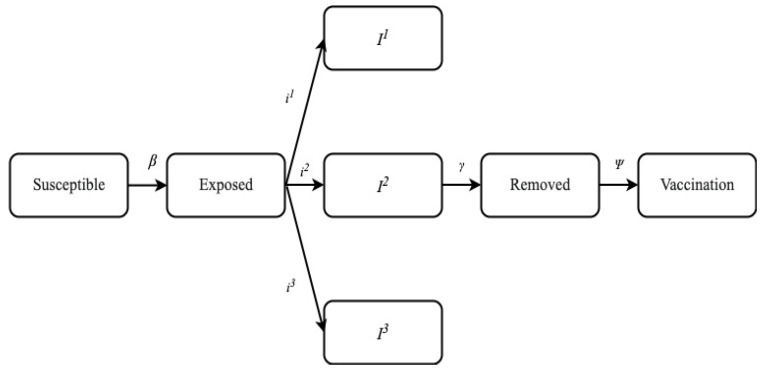
Proposed Model. *Ψ*—The rate at which individuals are vaccinated, *γ*—Rate at which infected individuals in class *I*^1^, *I*^2^ and *I*^3^ recovered from the disease and immunity is developed, *β*—Rate at which one infected in class *I*^1^, *I*^2^ and *I*^3^ contact susceptible and infect all of them. Thus, the susceptible individuals changed to exposed individuals, *I*^1^—Rate of mild infection and hospitalization.

**Figure 2:**
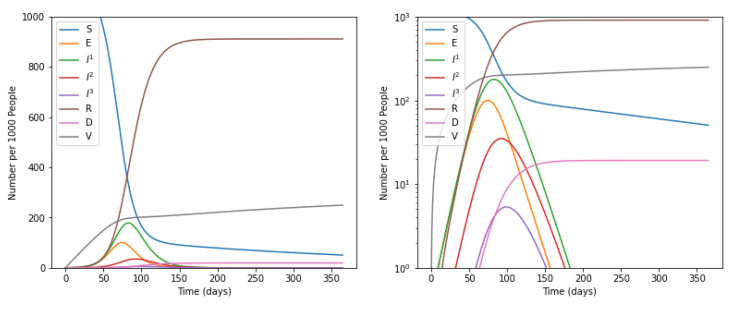
Trends of number of people in different categories over a period of 365 days without social distancing and vaccination. Susceptible individuals (*S*), Exposed individuals (*E*), Individuals who have recovered from the disease and have become immune (*R*), Dead individuals (*D*), and Vaccinated individuals (*V*), Individuals suffer with mild infection who do not require hospitalization (*I*^1^), Individuals with severe infection who require hospitalization (*I*^2^), Individuals with critical infection who require admittance to ICU (*I*^3^).

**Figure 3:**
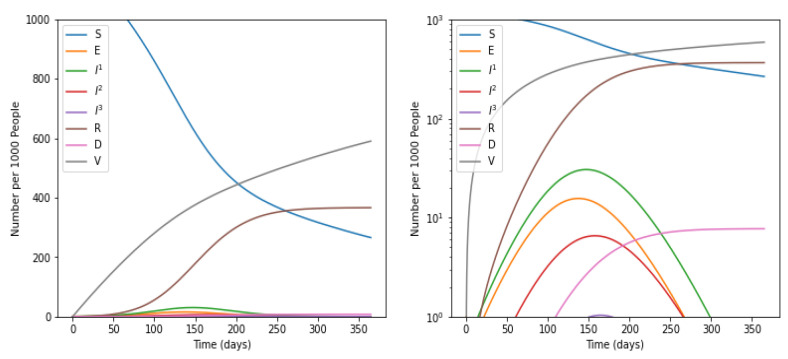
Impact of Social Distancing on number of cases in various categories. Susceptible individuals (*S*), Exposed individuals (*E*), Individuals who have recovered from the disease and have become immune (*R*), Dead individuals (*D*), and Vaccinated individuals (*V*), Individuals suffer with mild infection who do not require hospitalization (*I*^1^), Individuals with severe infection who require hospi-talization (*I*^2^), Individuals with critical infection who require admittance to ICU (*I*^3^).

**Figure 4:**
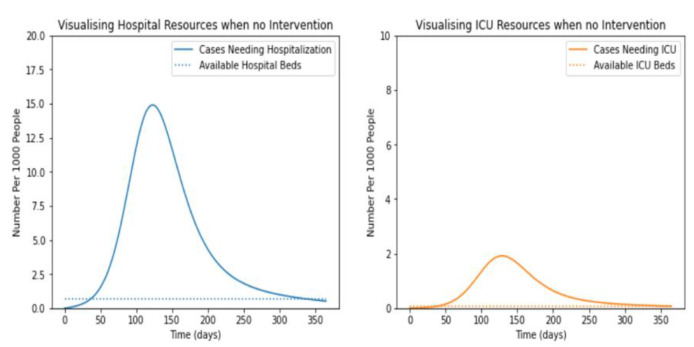
Impact of social distancing and vaccination.

Figure 1 showed the proposed model and sensitivity analysis of α (governmental policy action) and k (strength of public action) as shown in Figure 2, Figure 3 and Figure 4. It was observed from the analysis that to combat the pandemic, both governmental policy actions and public perception of risk was required. Further, the early approach of the peak or delaying the peak depended upon the healthcare capacities and management capabilities of a densely populated country such as India. For example, it was found empirically that when both governmental control measures (α = 0.8) and fair strength of public perception of risk (k = 1000) exist, in such a scenario the curve for the daily active cases would flatten out around 200 days from the start of the epidemic, as shown in Figure 4. The main objective behind such an analysis was to contribute to the conceptual understanding of the proposed mathematical model and demonstrate the extent of the impact of government policy measures and public perception of risk on the progress of the highly infectious disease COVID-19.

Rene Markovic et al. [22] presented a model for social networks that considered the heterogeneity of the population and different vaccination strategies. They proposed an agent-based epidemiological model, which offered some advantages compared to standard aggregate S (E) IR-type models, such as the inclusions of complex interaction patterns, locality of social contacts, and spatial heterogeneity of the population. Noteworthy, in our simulations, the latter has proven to be an essential factor affecting the trajectories of COVID-19 epidemics, particularly after the onset of vaccination and when the fraction of individuals with low health status in the population is relatively high.

A Mahata et al. [23], a fractional order dynamical system of susceptible, exposed, infected, recovered, and vaccinated populations was shown, with a single delay added in the infectious population to account for the time necessary for the said population to recover. They used the Adam–Bashforth–Moulton approach to derive numerical solutions to the model system.

Jiang et al. [24], suggested a machine learning algorithm with two modified SEIR models customized for the 2019-nCoV virus and vaccine uses to simulate the spread of COVID-19 in the UK (from January 2020 to March 2021) and made predictions of future cases. With different machine learning techniques, this work aimed to present enhanced SEIR models capable of a more accurate simulation for COVID-19 modelling and estimate.

Enrique and Ana [25], presented a vaccination model of the distribution of COVID-19. In this model, several optimization techniques are tested. The proposed model was tested for Argentina. It featured an essential demonstration of the impact of optimized vaccine distribution.

Anyin et al. [26] had researchers review and modelled age-structured cases, vaccination coverage, and vaccine BTI data from the Israeli Ministry of Health to better understand the epidemiological parameters involved in the epidemic. They developed a mathematical model that accounted for various characteristics, including age structure, vaccination efficiency over time, transmission rate over time, BTIs, vaccinated people’s lower susceptibility and infectivity, vaccine-induced immune protection duration, and vaccine distribution.

Nana-Kyere et al. [27] developed SEQIAHR compartmental model of COVID-19 to provide insight into the dynamics of the disease by underlying tailored strategies designed to minimize the pandemic. The model utilized the Castillo-Chavez method and Lyapunov function to investigate the global stability of the disease at the disease-free and endemic equilibrium.

Basic prediction modelling using time series data using SVM was completed by Singh et al. [28]. This study aimed to investigate the Corona Virus Disease 2019 (COVID-19) prediction of confirmed, deceased, and recovered cases. This prediction would help plan resources, determine government policy, provide survivors with immunity passports, and use the same plasma for care.

Initially, investigations were performed by Vaibhav et al. [29]. The primary investigation stated that age is not a significant factor that affected a person with this disease. Furthermore, the age attribute was normally distributed in the current dataset. A significant relationship was found between gender (male and female) and transmission type (imported from another country or communicated from local) of the patients. This study was carried out for the Indian scenario.

Continuing with the same analysis, Kumari et al. [30] presented a detailed study of recently developed forecasting models and predicted the number of confirmed, recovered, and death cases in India caused by COVID-19. The correlation coefficients and multiple linear regression applied for prediction and autocorrelation and autoregression have improved the accuracy.

Prominent work has been completed by Kou et al. [31], in which they predict a model for bankruptcy. They used a two-stage multi-objective feature selection method and compared it with different standard methods. Another study was completed by Liu et al. [32] in the field of economic policy, where the GARCH-MIDAS model was applied for evaluating the impact of different EPU indexes on the price volatility of European Union Allowance, and it was found that the accuracy of the EU EPU index was significantly higher than that of the global EPU index. Li had completed bibliometric work and Zu [33] presented the burst detection analysis of cited authors, journals, and references. At last, they not only reviewed the study of FinTech documents, but helped different scholars. A study has completed regarding the decision making of the financial market by Xiao and Ke [34]. At a glance, they presented current applications of deep learning, reinforcement learning, and fuzzy theory on the decision-making of financial markets.

A complete book has been published on a similar topic by Agarwal et al. [35]. This book discussed different mathematical analyses of infectious diseases, especially regarding COVID-19. Another significant work was completed by Otaki et al. [36]. They checked the distributions of SARS-CoV-2 non-self-mutation in the Omicron and Delta variants. The Machine Learning model was applied into the SIR model by Vega et al. [37]. They have applied SIMLR model for USA and Canada region. They have achieved good MAPE in comparison of current available models. This model can widely be used by other infectious disease.

### Novelty of the Proposed Research Work

After an analysis of related work was completed in this field, it was observed that the latest update in SIR model was the SEIRV model. This model included the term “vaccination” in the pandemic. In this research work, the existing model was enhanced by including the different parameters of infection by which the patient is suffering. These parameters are *I*^1^, *I*^2^, and *I*^3^. As such, the new model proposed was SEI (*I*^1^, *I*^2^, and *I*^3^) RV model. The *I*^1^, *I*^2^, and *I*^3^ were individuals who suffer with mild infection who do not require hospitalization (*I*^1^), individuals with severe infection who require hospitalization (*I*^2^), and individuals with critical infection who require admittance to ICU (*I*^3^). The infection rates were represented as $\beta^{1}$, $\beta^{2}$, and $\beta^{3}$. The proposed model was derived for COVID-19 cases.

## 4. Proposed Method

In this research, the SEIR model was extended to include eight parameters viz. the number of susceptible individuals (*S*), exposed individuals (*E*), individuals suffer with mild infection who do not require hospitalization (*I*^1^), individuals with severe infection who require hospitalization (*I*^2^), individuals with critical infection who require admittance to ICU (*I*^3^), individuals who have recovered from the disease and have become immune (*R*), dead individuals (*D*), and vaccinated individuals (*V*). The design of the proposed model is shown in Figure 1. The proposed model has five major compartments viz. susceptible, exposed, infected, removed, and vaccination.

The infected compartment was divided into three further sub compartments viz. *I*^1^ (where no severity and no hospitalization was required), *I*^2^ (there was some severity and patients hospitalized), and the last sub compartment was infection *I*^3^ (there was severity and patients were hospitalized in ICU). Similarly, the transmission rate was divided with $\beta^{1}$, $\beta^{2}$, and $\beta^{3}$.

Now, various coefficients were defined for simulating the SEIR model. These coefficients are described in Table 4. The proposed model is shown in Equations (6) to (13):

(6) $$\;\;\;\;S=-\beta^{1}I^{1}S-\beta^{2}I^{2}S-\beta^{3}I^{3}S\;$$

(7) $$E=\beta^{1}I^{1}S+\beta^{2}I^{2}S+\beta^{3}I^{3}S-aE+\eta V\left(\beta^{1}I^{1}+\beta^{2}I^{2}+\beta^{3}I^{3}\right)-\lambda E$$

(8) $$\;\;\;\;\;I^{1}=aE-\gamma^{1}I^{1}-p^{1}I^{1}-\lambda I^{1}\;\;$$

(9) $$\;\;\;\;I^{2}=p^{1}I^{1}-\gamma^{2}I^{2}-p^{2}I^{2}-\lambda I^{2}\;\;\;\;$$

(10) $$I^{3}=p^{2}I^{2}-\gamma^{3}I^{3}-\mu I^{3}-\lambda I^{3}\;\;\;$$

(11) $$R=\gamma^{1}I^{1}+\gamma^{2}I^{2}+\gamma^{3}I^{3}-\lambda R\;\;\;\;$$

(12) $$D=\mu I^{3}\;\;$$

(13) $$V=S\psi -\eta V\left(\beta^{1}I^{1}+\beta^{2}I^{2}+\beta^{3}I^{3}\right)-\lambda V\;\;$$

The total population comprising 1000 individuals is the sum of all individuals who are part of one of the above said categories, as shown in Equation (14).

*N*_0_ = *S* + *V* + *E* + *I*^1^ + *I*^2^ + *I*^3^ + *R* + *D* (14)

Now, the epidemic of COVID-19 is simulated within a population. The simulation is completed on the Geographical location of USA. The tailored SEIR model is employed to trace the impact of vaccination and social distancing on the rate of susceptibility, exposure, infection, and recovery. Furthermore, the impact of vaccination and social distancing have been analyzed to identify the time delay in achieving the peak of disease severity. Further, the impact of these parameters on the hospital resource requirement have been analyzed. The values of parameters, such as incubation period, duration of mild infection, fraction of population with mild, severe, and critical infection, rate of fatality, duration from ICU hospitalization to death, duration of hospitalization, vaccination rate, vaccination inefficacy, birth rate, and natural death rate is preset for the purpose of experiments. The reproduction *R*_0_ is Equations (15) and (16):

(15) $$\;\;\;\;\;\;\;\;\;\;\;\;R_{0}=N_{0}*\frac{1}{\left(\lambda \right)}\frac{1}{\left(\lambda +\psi \right)}\left(\frac{\beta^{1}}{\left(p^{1}+\gamma^{1}+\lambda \right)}+\frac{p^{1}}{\left(p^{2}+\gamma^{1}+\lambda \right)}\left(\frac{\beta^{2}}{\left(p^{2}+\gamma^{2}+\lambda \right)}+\frac{p^{2}}{\left(p^{2}+\gamma^{2}+\lambda \right)}*\frac{\beta^{1}}{\left(\mu +\gamma^{3}+\lambda \right)}\right)\right)\left(\lambda +\psi *\eta \right)*\sigma \;$$

(16) $$\;\;\;\;\;\;\;\;\;\;\;\;R_{0}=N_{0}*\frac{1}{\left(\lambda \right)}\frac{1}{\left(\lambda +\psi \right)}\left(\frac{\beta^{1}\text{slow}}{\left(p^{1}+\gamma^{1}+\lambda \right)}+\frac{p^{1}}{\left(p^{2}+\gamma^{1}+\lambda \right)}\left(\frac{\beta^{2}\text{slow}}{\left(p^{2}+\gamma^{2}+\lambda \right)}+\frac{p^{2}}{\left(p^{2}+\gamma^{2}+\lambda \right)}*\frac{\beta^{3}\text{slow}}{\left(\mu +\gamma^{3}+\lambda \right)}\right)\right)\left(\lambda +\psi *\eta \right)*\sigma \;$$

where:

(17) $$\beta^{1}slow=0.6*\beta^{1},\;\beta^{2}slow=0.6*\beta^{2},\;\beta^{3}slow=0.6*\beta^{3}$$

σ = Crude Birth Rate.

## 5. Result and Discussion

The main objective of this paper was to develop a mathematical model that can predict the cases of COVID-19. A new fifth compartment was added in the base of SEIR. The effect of the proposed model was simulated on three aspects: the first aspect was when social distancing was not incorporated; the second was when social distancing was incorporated; and the third was when social distancing was incorporated when population was vaccinating.

### 5.1. Without Intervention of Social Distancing

Initially, the number of people of various categories viz, susceptible, exposed, infected at mild, severe, or critical stage, recovered, dead, and vaccinated were visualized over a period of 365 days. The trends of increasing or decreasing the number of people in each of the above-stated categories without considering the impacts of vaccination and social distancing is shown in Figure 2. While visualizing the number of people in each category, the calculated values of coefficients β, α, γ, p, μ corresponding to σ, λ, η, ψ, respectively, were used. These values are shown in Table 5.

By using the above stated parameters, the proposed model reported the value of $R_{0}$ as 2.1619, without considering the impact of social distancing and vaccination. Furthermore, the model predicted an epidemic growth rate of about 0.06 per day and the number of infected people doubled after every 10.7 days. Moreover, it is evident from the graph shown in Figure 1 that there was a slight decrease in the number of susceptible people from 0 to 100 days of infection. The number of susceptible people became minimum between 100 to 150 days. This number again shows a slight increase after 150 days and became constant. Simultaneously, the number of people exposed, infected at mild, severe, or critical stage increases at a sharp rate and became maximum in the duration of 100 to 150 days.

### 5.2. With Intervention of Social Distancing

Social distancing norms and vaccination were considered to further simulate the spreading of COVID-19. Now, the model reported the value of *R*_0_ as 1.3, and an epidemic growth rate of 0.01 per day. A significant decrease of 0.8619 was observed in the value of *R*_0_. Further, the doubling rate for the number of infected cases increased to 54.5 days.

### 5.3. Impact of Social Distancing and Vaccination on the Number of Infectious Cases

It has been observed that the infected individuals at severe and critical stages required hospitalization. Based on the severity of the infection, they needed to be admitted to non-ICU or ICU units. To identify the impact of social distancing and vaccination on the requirement for hospitalization, the experiments were performed. The results shown in Figure 4, demonstrated the requirements and availability of non-ICU and ICU beds. 

**Figure 5:**
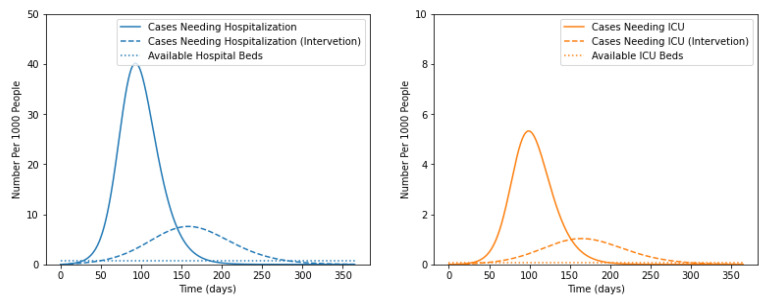
Compare to the cases with intervention.

It was evident from the graph shown in Figure 5 that the number of non-ICU and ICU beds would be exhausted after 37.4 and 38.1 days, respectively. Further, it was clear from the figure that non-ICU beds’ requirement reached its maximum from 70 to 120 days of infection. This may be due to the transformation of mild to severe cases. Similarly, the requirements for ICU beds are maximum from 100 to 150 days. This may be caused by the transformation of severe to critical patients.

**Figure 6:**
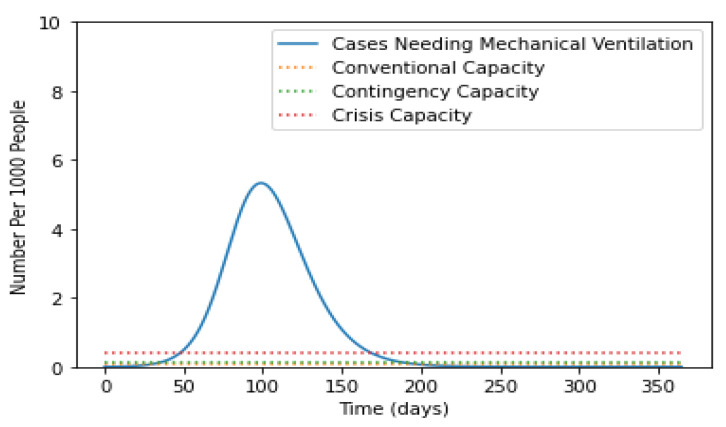
Trends of ventilation support.

Further, patients with severe lung infections required mechanical ventilation support. The trends of ventilation support are demonstrated in Figure 6. Moreover, the requirements of ventilation support in convention, contingency, and crisis were demonstrated.

Now on observing the fact that only severe or critical cases go to the hospital and all critical cases require ICU care and mechanical ventilation, we observed the following graph.

This means the susceptible people were continuously transforming to infected people. In addition, the number of deaths also reached its maximum between the period of 100 to 150 days. In strong contrast, it was evident from the graph shown in Figure 2 that there was a sharp decrease in the number of sensitive cases with a simultaneous rise in vaccination rates and recovered individuals. Additionally, there was a sharp decrease in the number of infected individuals when the number of vaccinations achieved its maximum rate. Moreover, the number of deaths declined at a sensitive rate with an increase in vaccinations and recovered people. It was apparent from the graph shown in Figure 3 that there was a sharp fall in susceptible and infected individuals. Merely a few people lie in the category of mild infections.

Moreover, the number of individuals in severe and critical categories became negligible, and the number of deaths reached its minimum. Based on the above discussion, it was clear that following the social distancing norms and vaccination drive played a significant role in beating COVID-19. Furthermore, it provided more time to prepare the health industry for dealing with the pandemic of COVID-19.

## 6. Conclusions

Everyone is now affected by COVID-19 as it is a global pandemic. Contemporary models are one of the authentic models for predicting the spread of a pandemic disease. In this paper, different pandemic models are studied, such as SIR, SEIR, SEIRD, and SEIRV models, etc. All these models fit in various types of diseases. Vaccination is found to be one of the best solutions to mitigate the spread of the COVID-19 pandemic. A new SEIRV model is proposed that considers the vaccination rate. In this model, different compartments are included, such as severity of the patient, vaccination rate, death rate, and birth rate. Without imparting social distancing, the proposed model obtained the value of R_0_ is 2.1619. The proposed model is mathematically simulated and tested in different situations, such as without intervention of social distancing, and with the intervention of social distancing. The model predicted an epidemic growth rate of about 0.06 per day, and the number of infected people doubled after every 10.7 days. By imparting social distancing, the proposed model obtained the value of R_0_ is 1.3. Finally, it can be inferred that social distancing norms and vaccination drive play a significant role in overcoming COVID-19. In the future, the proposed model will be enhanced by incorporating new compartments, such as vaccination of infants and kids, hospitalization, etc.

## Figures and Tables

**Table 1:** Vaccines Approved by WHO.

S. N.	Name of Company	Name of Vaccine
1	Moderna	mRNA-1273
2	Pfizer/BioNTech	BNT162b2
3	Janssen (Johnson & Johnson)	Ad26.COV2.S
4	Oxford/AstraZeneca	AZD1222
5	Serum Institute of India	Covishield
6	Bharat Biotech	Covaxin
7	Sinopharm (Beijing)	BBIBP-CorV (Vero Cells)
8	Sinovac	CoronaVac

**Table 2:** Country-wise vaccination rates.

S. N.	Name of Country	% of Population Fully Vaccination
1	India	37.3%
2	United States	60.8%
3	Brazil	65.6%
4	Indonesia	37.7%
5	Japan	77.9%
6	Russia	42.6%
7	Germany	69.5%
8	United Kingdom	69.5%
9	France	71.0%
10	Iran	57.3%
11	Saudi Arabia	65.5%
12	Egypt	16.4%
13	South Africa	25.8%
14	United Arab Emirates	91.1%
15	Nigeria	1.9%

**Table 3:** Different compartment models.

S. N.	Acronym	Name of Model	Parameter Added	Definition
1	SIS [7]	Susceptible-Infectious-Susceptible	Simplest form	Immunity does not build
2	SIRD [8]	Susceptible-Infectious-Recovered-Deceased	Deceased	D is the mortality rate
3	MSIR [9]	Maternal-Susceptible-Infectious-Recovered	Maternally Derived Immune	Newborn babies which are immune to a specific disease, such as measles
4	SICR [10]	Susceptible-Infectious-Carrier-Recovered	Carrier	It is applicable on those where infection resides in the body forever, such as TB
5	SUQC [11]	Susceptible-Unquarantined, Quarantine-Confirmed	Unquarantined, Quarantine	Number of people who are quarantined and unquarantined.
6	GSIR [12]	Generalized-Susceptible-Infectious-Recovered	Generalized	Assumed that throughout time, many waves of varied peak amplitude and form arise and fade away
7	SEIHR [13]	Generalized-Susceptible-Infectious-Hospitalized-Recovered	Hospitalized	Number of persons hospitalized
8	SCEIR [14]	Susceptible-Exposed-Infectious-Recovered-Removed	Confined	When an individual is experiencing lockdown
9	ISSEIR [15]	Interacting Subpopulation- Susceptible-Exposed-Infectious-Recovered	Interacting Subpopulation	Separate SEIR model between each subgroup of the population
10	SEIRV [16]	Susceptible-Infectious-Recovered-Vaccination	Vaccination	When the population is vaccinated

**Table 4:** Coefficient of the proposed methods.

Name of Coefficient	Definition
$N_{0}$	Total population (comprising 1000 individuals in this research)
*S*	Susceptible individuals
$\beta^{1}$	Rate at which one infected in class *I*^1^ contact susceptible and infect all of them. Thus, the susceptible individuals changed to exposed individuals.
$\beta^{2}$	Rate at which one infected in class *I*^2^ contact susceptible and infect all of them. Thus, the susceptible individuals changed to exposed individuals.
$\beta^{3}$	Rate at which one infected in class *I*^3^ contact susceptible and infect all of them. Thus, the susceptible individuals changed to exposed individuals.
*I* ^1^	Rate of mild infection and hospitalization not required.
*I* ^2^	Rate of severe infection and hospitalization is required.
*I* ^3^	Rate of critical infection and I.C.U. is required.
*E*	Set of exposed individuals; they are infected but not asymptotic and infectious.
*V*	Set of vaccinated persons.
$\gamma^{1}$	Rate at which infected individuals in class *I*^1^ recovered from the disease and immunity is developed.
$\gamma^{2}$	Rate at which infected individuals in class *I*^2^ recovered from the disease and immunity is developed.
$\gamma^{3}$	Rate at which infected individuals in class *I*^3^ recover from the disease and immunity is developed.
*p* ^1^	Rate at which one infected in class *I*^1^ is shifted to class *I*^2^_._
*p* ^2^	Rate at which one infected in class *I*^2^ is shifted to class *I*^3^_._
*R*	Set of individuals who have recovered from the disease and are now immune.
λ	Rate of natural (those who are not deceased from the COVID-19).
μ	The death rate of individuals in the most severe stage of disease.
Ψ	The rate at which individuals are vaccinated
η	Vaccine inefficacy
*D*	Set of removed populations

**Table 5:** Obtained values of coefficients.

Coefficient	Crude Birth Rate (σ)	λ	η	ψ
β	0.0	0.00025	0.0	0.0
α	0.2	-	-	-
*γ*	0.0	0.08	0.06818182	0.08571429
p	0.0	0.02	0.02272727	-
μ	0.057142857	-	-	-

## Data Availability

The data presented in this study are available on request from the corresponding author.
